# Group II Intron-Based Gene Targeting Reactions in Eukaryotes

**DOI:** 10.1371/journal.pone.0003121

**Published:** 2008-09-01

**Authors:** Marta Mastroianni, Kazuo Watanabe, Travis B. White, Fanglei Zhuang, Jamie Vernon, Manabu Matsuura, John Wallingford, Alan M. Lambowitz

**Affiliations:** Institute for Cellular and Molecular Biology, University of Texas at Austin, Austin, Texas, United States of America; Yale University, United States of America

## Abstract

**Background:**

Mobile group II introns insert site-specifically into DNA target sites by a mechanism termed retrohoming in which the excised intron RNA reverse splices into a DNA strand and is reverse transcribed by the intron-encoded protein. Retrohoming is mediated by a ribonucleoprotein particle that contains the intron-encoded protein and excised intron RNA, with target specificity determined largely by base pairing of the intron RNA to the DNA target sequence. This feature enabled the development of mobile group II introns into bacterial gene targeting vectors (“targetrons”) with programmable target specificity. Thus far, however, efficient group II intron-based gene targeting reactions have not been demonstrated in eukaryotes.

**Methodology/Principal Findings:**

By using a plasmid-based *Xenopus laevis* oocyte microinjection assay, we show that group II intron RNPs can integrate efficiently into target DNAs in a eukaryotic nucleus, but the reaction is limited by low Mg^2+^ concentrations. By supplying additional Mg^2+^, site-specific integration occurs in up to 38% of plasmid target sites. The integration products isolated from *X. laevis* nuclei are sensitive to restriction enzymes specific for double-stranded DNA, indicating second-strand synthesis via host enzymes. We also show that group II intron RNPs containing either lariat or linear intron RNA can introduce a double-strand break into a plasmid target site, thereby stimulating homologous recombination with a co-transformed DNA fragment at frequencies up to 4.8% of target sites. Chromatinization of the target DNA inhibits both types of targeting reactions, presumably by impeding RNP access. However, by using similar RNP microinjection methods, we show efficient Mg^2+^-dependent group II intron integration into plasmid target sites in zebrafish (*Danio rerio*) embryos and into plasmid and chromosomal target sites in *Drosophila melanogster* embryos, indicating that DNA replication can mitigate effects of chromatinization.

**Conclusions/Significance:**

Our results provide an experimental foundation for the development of group II intron-based gene targeting methods for higher organisms.

## Introduction

Mobile group II introns employ a remarkable site-specific DNA integration mechanism, termed retrohoming, in which the excised intron RNA inserts directly into a DNA target site and is then reverse transcribed by the intron-encoded protein (IEP) [1], [2]. This process is mediated by a ribonucleoprotein particle (RNP), which is formed during RNA splicing and contains the IEP and excised intron lariat RNA. RNPs initiate DNA integration by recognizing DNA target sites, with most of the target sequence recognized by base pairing of the intron RNA and only a small number of bases recognized by the IEP [3]–[7]. This feature together with their high insertion frequencies and specificity enabled the development of mobile group II introns into novel bacterial gene targeting vectors (“targetrons”), which can be programmed to insert efficiently into desired DNA targets simply by modifying the intron RNA [8]. Targetrons have been used to obtain gene knock-outs and knock-ins in diverse Gram-negative and Gram-positive bacteria, including medically and commercially important species in which gene targeting by other methods had previously been difficult or impossible [9]–[15]. Thus far, however, it has not been shown that targetrons can function efficiently in eukaryotic cells.

The *Lactococcus lactis* Ll.LtrB intron has been used as a model system both to study group II intron mobility and to develop gene targeting applications [1]. Ll.LtrB encodes a protein, denoted LtrA, with four conserved domains: RT, which corresponds to the fingers and palm regions of retroviral RTs; X, which corresponds at least in part to the RT thumb; DNA-binding (D); and DNA endonuclease (En). The RT and X domains bind specifically to the intron RNA to stabilize the catalytically active RNA structure for RNA splicing and reverse splicing, while the D and En domains interact with DNA target sites during intron mobility. For DNA target site recognition, the intron RNA base pairs to DNA target site positions -12 to +2 (numbered from the intron-insertion site), while the IEP recognizes a small number of nucleotide residues in the flanking 5′- and 3′-exon regions. The IEP interactions in the distal 5′-exon lead to local DNA melting, enabling the intron RNA to base pair to a target sequence in one DNA strand, while the IEP interactions with the 3′ exon are required for En-domain cleavage between positions +9 and +10 of the opposite strand. The 3′ DNA end generated at the cleavage site is then used by the IEP as a primer for reverse transcription of the inserted intron RNA, resulting in an intron cDNA that is integrated by host enzymes [16], [17]. Unlike retroviral RTs, the LtrA protein lacks an RNase H domain for degradation of the intron RNA template strand and has very low processive DNA-dependent DNA polymerase activity for second-strand synthesis, necessitating the use of host enzymes for these functions [17].

For gene targeting in bacteria, the Ll.LtrB intron is introduced via a donor plasmid, which expresses an Ll.LtrB-ΔORF intron with flanking exons, followed by the LtrA protein from a position just downstream of the 3′ exon [4], [8]. The intron is targeted to desired sites with the aid of a computer algorithm, which scans the target sequence for the best matches to the positions recognized by the IEP and then designs PCR primers for modifying the intron to base pair optimally to those sites [7]. The positions recognized by the IEP are sufficiently few and flexible that the algorithm generally identifies multiple rank-ordered target sites in any gene. Further, the intron can be targeted to insert in different orientations by targeting one or the other DNA strand, making it possible to obtain either unconditional or conditional disruptions, depending on whether the intron is in the correct orientation to be spliced from precursor RNA by providing the LtrA protein [8], [9], [11]. Selectable markers can be incorporated into the intron, but because integration frequencies are usually high, the desired integrants are often identified simply by colony PCR screening without selection [7].

It would be highly desirable to develop analogous group II intron-based gene targeting methods for higher organisms. With the exception of mouse embryonic stem cells and a few other cell types, mammalian cells and those of other higher eukaryotes lack efficient homologous recombination systems for gene targeting [18], [19]. Although RNAi has been a major advance in genetic manipulation in higher organisms, it yields “knock downs” rather than “knock outs”, requires continuous expression or repeated introduction, has off-target effects, and does not permit the introduction of new genes [20]–[22]. Currently available gene therapy vectors, either do not integrate stably (adenovirus) or insert at multiple, semi-random DNA sites with possible deleterious effects (retroviruses, rep-deficient adeno-associated virus; AAV) [23]–[27].

To overcome such deficiencies, alternative gene targeting methods are being developed, employing different means to increase the frequency of homologous recombination. One of the best developed of such methods for human cells, homologous recombination with recombinant AAV, generally yields 0.1–15% of integrations at the desired target site [28], [29]. Other methods with potentially higher specificity involve the use of site-specific DNA endonucleases, such as meganucleases or artificial Zn-finger nucleases, to make a targeted double-strand break that promotes homologous recombination [30]–[33]. Recently, efficient gene targeting in mammalian cells has been achieved by using Zn-finger nucleases in this way to stimulate homologous recombination with a co-transformed donor DNA [34]–[36]. This method has been reported to give recombination frequencies up to 29% in mammalian cells and is efficient enough to target both alleles in diploids. Zn-finger nucleases have also been used to obtain gene disruptions in mammalian cells by incorrect nonhomologous end-joining [37] and are being applied for gene targeting in plants and eukaryotic model organisms [38]–[42]. Ultimately, the utility of this and other methods will depend on the ease of designing and optimizing protein endonucleases to recognize different DNA target sequences, their specificity and toxicity, and their cost and availability compared to other gene targeting methods.

Although still undeveloped for use in eukaryotes, mobile group II introns can potentially be employed in the same way as Zn-finger nucleases and meganucleases to introduce a targeted double-strand break that stimulates homologous recombination or yields mutations by inaccurate non-homologous end-joining [8]. In the case of group II introns, the double-strand break results from the initial reverse splicing and second-strand cleavage reactions. Reverse splicing occurs in two steps, the first of which is attachment of intron lariat RNA to the 5′ end of the 3′ exon leaving a strand break, while second-strand cleavage is a conventional DNA endonuclease reaction catalyzed by the IEP [1]. Compared to protein endonucleases, group II introns offer the potential advantage that the double-strand break can be targeted to different locations simply by modifying the base pairing sequences in the intron RNA, thereby obviating the need for protein engineering to change target specificity [8]. Mobile group II introns, like retroviruses to which they are related, can also be used as integrating vectors, enabling the insertion of cargo genes at desired sites, with the advantage of high, controllable DNA target specificity [5], [9], [43].

Previously, we retargeted the Ll.LtrB group II intron to insert in the HIV-1 provirus and the human gene encoding the HIV-1 co-receptor CCR5 and showed that the retargeted introns insert into their target sites with high frequency and specificity in *Escherichia coli* plasmid assays [4]. We also used *E. coli* assays to show that group II introns could be used for genetic repair, either by inserting a functional copy of the gene into a defective gene, or by inserting functional exons preceded by a splice acceptor site to circumvent defective downstream exons [43]. Such retargeted group II intron RNPs retained activity in human cell assays in which plasmid-borne target sites and group II intron RNPs were introduced separately by liposome-mediated transfection [4]. In these initial experiments, however, group II intron integration into plasmid target sites in human cells was much less efficient than in bacteria, requiring nested PCR for detection.

There was reason to believe that group II introns might be particularly sensitive to low Mg^2+^ concentrations in eukaryotic cells. In the yeast *Saccharomyces cerevisiae*, mutations in mitochondrial Mg^2+^-transport proteins, which result in decreased intramitochondrial Mg^2+^ concentrations, strongly inhibited the splicing of all four mtDNA group II introns, while having much less effect on the splicing of group I introns [44], [45]. Standard *in vitro* reaction conditions for the Ll.LtrB intron use 5 mM Mg^2+^ for IEP-promoted RNA splicing and 10 mM Mg^2+^ for reverse splicing of RNPs into DNA target sites [46]. Thus, it is possible that group II intron RNPs are unable to function optimally in eukaryotic nuclei where the free Mg^2+^ concentration is estimated to be 1–2 mM [47].

Here, we used a *Xenopus laevis* oocyte microinjection assay to show that group II intron RNPs can insert efficiently into a plasmid target site in eukaryotic nuclei, but require the injection of additional MgCl_2_. In such assays under optimal conditions, group II intron RNP integration frequencies were as high as 38% and targeting frequencies by double-strand break- stimulated homologous recombination were as high as 4.8%. Analogous RNP microinjection assays show relatively efficient Mg^2+^-dependent group II intron integration into plasmid target sites in *D. melanogaster* and zebrafish (*Danio rerio*) embryos and into chromosomal target sites in *D. melanogaster* embryos. Our results demonstrate the potential for using group II introns for gene targeting reactions in higher organisms.

## Results

### Site-specific group II intron integration into plasmid target sites in *X. laevis* oocyte nuclei

**Figure 1:**
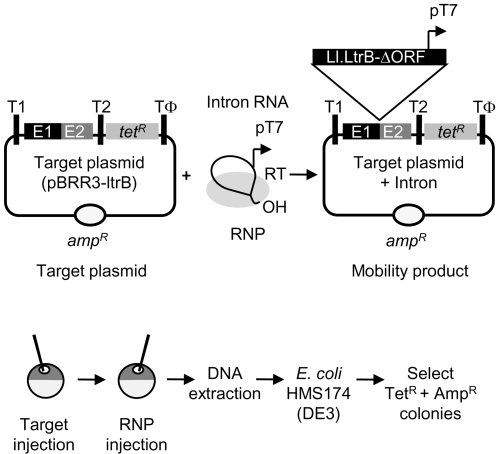
Group II intron-integration assay in *X. laevis* oocyte nuclei. (A) Plasmid assay. Microinjected group II intron RNPs containing a 0.9-kb Ll.LtrB-ΔORF intron RNA with a T7 promoter in DIV and the group II intron RT integrate into a target site (ligated *ltrB* exon 1 and 2 sequences; E1 and E2) cloned upstream of a promoterless *tet^R^* gene in an Amp^R^ target plasmid (pBRR3-ltrB), thereby activating the *tet^R^* gene. T1 and T2 are *E. coli rrnB* transcription terminators, and Tφ is a phage T7 transcription terminator. (B) Protocol. Target plasmids and RNPs were injected into oocyte nuclei using different needles. After incubating the oocytes for different times, nucleic acids were isolated and electroporated into *E. coli* HMS174(DE3). The integration efficiency was calculated as the ratio of (Tet^R^+Amp^R^)/Amp^R^ colonies.

To assay group II intron targeting reactions in *X. laevis* oocyte nuclei, we used a plasmid assay patterned after one developed to assay group II intron mobility in *E. coli* (Fig. 1A) [4], [8]. In the *E. coli* assay, an Ll.LtrB intron with a phage T7 promoter inserted near its 3′ end integrates into a target site (the ligated E1-E2 sequence of the *ltrB* gene) cloned upstream of a promoterless *tet^R^* gene in an Amp^R^ target plasmid. Insertion of the intron containing the T7 promoter into the target site activates the *tet^R^* gene, enabling intron-integration efficiencies to be measured by the ratio of (Tet^R^+Amp^R^)/Amp^R^ colonies. For gene targeting applications, we generally use a streamlined 0.9-kb derivative of the Ll.LtrB intron from which ORF sequences encoding the IEP have been deleted. This Ll.LtrB-ΔORF intron has much higher integration efficiency and nuclease-resistance than does the full-length intron [4].

The protocol used for the assays in *X. laevis* oocyte nuclei is shown in Figure 1B. The target plasmid (pBRR3-1trB) containing the *ltrB* target site was injected first, followed within 1 min by Ll.LtrB RNPs reconstituted with self-spliced Ll.LtrB-ΔORF lariat RNA and purified recombinant LtrA protein (see Materials and Methods). In all the *X. laevis* experiments, the DNA target plasmid and RNPs were injected using different needles to avoid prior mixing, and 10 or more oocytes were injected for each experimental condition. After incubation for times specified for individual experiments, the oocytes for each condition were pooled, and nucleic acids were extracted and electroporated into *E. coli* HMS174(DE3), which expresses phage T7 RNA polymerase. The cells were then plated on LB medium containing ampicillin with or without tetracycline to determine the mobility efficiency as described above.

As shown in Table 1, when the target plasmid DNA and intron RNPs were injected without further additions, no Tet^R^ colonies were obtained. In light of the previous studies indicating that group II introns are particularly sensitive to low Mg^2+^ concentrations (see Introduction), we repeated the experiment, now injecting the target plasmid in a solution containing 500 mM MgCl_2_. Under these conditions, the RNP integration efficiency increased dramatically to 27% in this experiment. We confirmed that the intron had integrated precisely at the expected target site by sequencing both the 5′- and 3′-integration junctions in plasmids isolated from seven independent Tet^R^ colonies (not shown), and controls showed no Tet^R^ colonies after heating the RNPs to 95°C for 3 min or using RNPs reconstituted with a mutant LtrA protein that lacks RT activity (RT^−^). The integration efficiency was proportional to the amount of RNPs injected (not shown) and with different batches of RNPs ranged up to 38% of plasmid target sites (with injection of 20 nl of 2.5 mg/ml RNPs). Finally, efficient integration was obtained only when both the RNP and target plasmid were injected into the nucleus and not when they were injected into the cytoplasm (Table 1). Together, these experiments show that under appropriate conditions, the site-specific group II intron-integration reaction can occur efficiently in eukaryotic nuclei.

**Table 1:** Site-specific integration of a group II intron into a plasmid target site in *X. laevis* oocyte nuclei.

Condition	Amp^R^+Tet^R^	Amp^R^	Integration (%)
RNP+TP	0	9.4×10^4^	<0.001
RNP+TP+MgCl_2_	4.8×10^3^	1.8×10^4^	27
Heated RNP+TP+MgCl_2_	0	5.5×10^4^	<0.002
RNP/RT^−^+TP+MgCl_2_	0	1.7×10^4^	<0.006
RNPs+TP+MgOAc	6.2×10^2^	1.5×10^4^	4.1
RNPs+TP+MgSO_4_	5.1×10^2^	1.8×10^4^	2.8
RNPs+TP+MnCl_2_	0	3.5×10^3^	<0.03
RNPs+TP+CaCl_2_	0	2.5×10^3^	<0.04
RNPs+TP+MgCl_2_ (cyto)	23	4.6×10^4^	0.05

Target plasmid pBRR3-ltrB (TP; 4.6 ng, 18 nl) and Ll.LtrB intron RNPs (32 ng, 16 nl) were injected separately into *X. laevis* oocyte nuclei. After incubating for 2 h at 25°C, nucleic acids were extracted from 10 pooled oocytes for each condition, electroporated into *E. coli* HMS174(DE3), and assayed for site-specific group II intron integration into the plasmid target site by determining the ratio of (Tet^R^+Amp^R^)/Amp^R^ colonies, as described in Figure 1 and Materials and Methods. In a control (row 3), RNPs were heated to 95°C for 3 min before injection. MgCl_2_, Mg acetate, MgSO_4_, MnCl_2_, and CaCl_2_ were injected at a concentration of 500 mM with the target plasmid. In all assays, 17 mM dNTPs was included with the target plasmid, except those for MnCl_2_ and CaCl_2_ due to the formation of precipitates. In one assay (bottom row), the RNPs and target plasmid plus MgCl_2_ and dNTPs were injected into the cytoplasm instead of the nucleus. The Table shows the integration efficiency determined by the average number of (Tet^R^+Amp^R^) and Amp^R^ colonies based on counting three plates at appropriate dilutions. Assays for all conditions were repeated at least once with similar results.

### Effect of Mg^2+^, dNTPs, and temperature on group II intron integration in *X. laevis* oocyte nuclei

**Figure 2:**
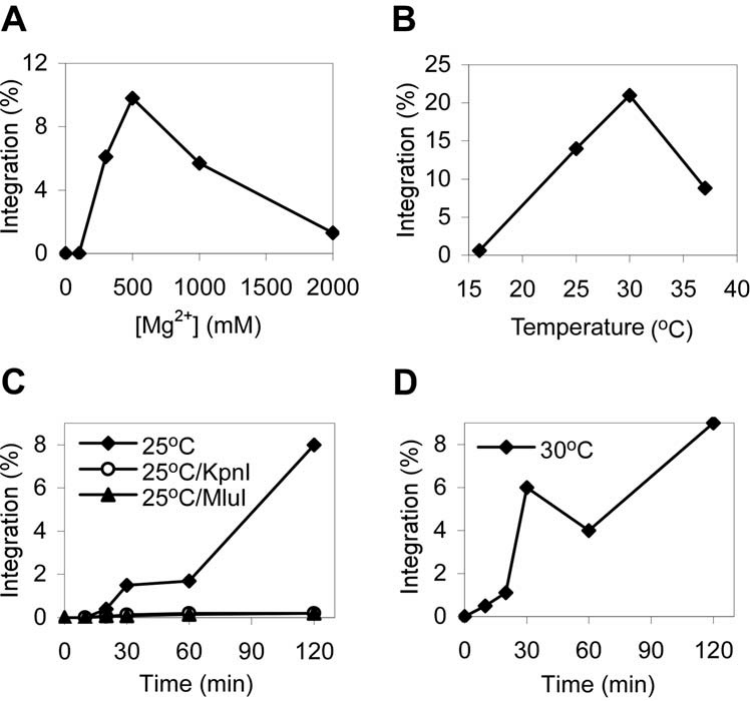
Determination of optimal conditions for site-specific integration of group II intron RNPs into a plasmid target site in *X. laevis* oocyte nuclei. (A) Mg^2+^-concentration dependence. Target plasmid DNA (10 ng) with 0 to 2,000 mM MgCl_2_ and 17 mM dNTPs was injected into *X. laevis* oocyte nuclei followed by Ll.LtrB lariat RNPs (50 ng). The oocytes were incubated for 2 h at 25°C. (B) Temperature dependence. Target plasmid DNA (9 ng) with 500 mM MgCl_2_ and 17 mM dNTPs was injected into *X. laevis* oocyte nuclei followed by Ll.LtrB lariat RNPs (50 ng). The oocytes were incubated for 2 h at different temperatures. (C and D) Time courses at 25°C or 30°C, respectively. Target plasmid DNA (4.5 ng) with 500 mM MgCl_2_ and 17 mM dNTPs was injected into *X. laevis* oocyte nuclei, followed by Ll.LtrB lariat RNPs (36 ng). The oocytes were incubated for the indicated times and then quick frozen on dry ice. Nucleic acids isolated from the 25°C-incubated oocytes were untreated or digested with KpnI or MluI (10 units; New England Biolabs) for 1 h at 37°C and then extracted twice with phenol-CIA and ethanol precipitated prior to electroporation into *E. coli*. In (A)–(D), injection volumes were 18 nl. Integration efficiencies (%) were determined by electroporating nucleic acids extracted from oocytes into *E. coli* HMS174(DE3), followed by plating to determine ratios of (Tet^R^+Amp^R^)/Amp^R^ colonies, as described in Figure 1 and Materials and Methods. Each experiment was repeated at least once with essentially the same results.

To determine the optimal injected Mg^2+^ concentration for group II intron integration, we measured the group II intron-integration efficiency at different MgCl_2_ concentrations injected in a volume of 18 nl with plasmid DNA. Figure 2A shows that the highest integration efficiencies were obtained when the injected Mg^2+^ concentration was 500 mM. Assuming that the oocyte has a volume of 1 µl and that all of the injected Mg^2+^ remains free, this injection would raise the intracellular Mg^2+^ concentration by ∼9 mM, close to the Mg^2+^ optimum for the DNA integration reaction [46]. MnCl_2_ or CaCl_2_ could not substitute for MgCl_2_, and Mg acetate or MgSO_4_ gave significantly lower intron-integration frequencies than did MgCl_2_ (Table 1). The order of injection of DNA and RNPs made no difference, and the Mg^2+^ could be injected separately from the DNA. However, high Mg^2+^ concentrations could not be injected with the RNPs because it caused them to aggregate, making double-injection necessary (not shown).

Studies with bovine capillary endothelial cells showed that the addition of 65 mM MgCl_2_ to the culture medium results in chromosome unfolding [48]. While this may also occur in *X. laevis* oocyte nuclei and could facilitate group II intron integration, we found that addition of 10–500 mM MgCl_2_ to the *X. laevis* oocyte incubation medium resulted in little or no increase in the group II intron-integration efficiency (the maximum was 4×10^−4^ % with 65 mM MgCl_2_).

In early experiments, there was considerable variation in the intron-integration efficiency between different batches of oocytes (insertion frequencies 0.002–7%). Later, we found that this problem could be mitigated by addition of 17–20 mM each of dATP, dCTP, dGTP, and dTTP (“dNTPs”) to the DNA/MgCl_2_ injection mixture, which increased group II intron-integration frequencies in suboptimal oocytes to >3% (not shown). Higher dNTP concentration did not further increase the integration frequencies. In later experiments, 17 mM dNTPs were added routinely.

In experiments done at optimal Mg^2+^ and dNTP concentrations, the highest integration efficiencies were obtained at temperatures of 25–30°C (Fig. 2B). Integration was barely detectable at 16°C and was inhibited somewhat at 37°C, possibly reflecting the deleterious effect of high temperature on the oocyte. Temperatures≥30°C are known to activate the heat-shock response and block transcription in *X. laevis* oocytes [49]. Based on the above experiments, 500 mM MgCl_2_, 17 mM dNTPs, and temperatures of 25 or 30°C were used routinely in the experiments below.

### Time-course of intron integration and nuclease sensitivity of retrohoming products

In time-course experiments at 25 or 30°C, retrohoming products appeared after 10 min and increased for at least 120 min, reaching maximum values of 8–10% in those experiments (Fig. 2C and 2D). No mobility products were detected when the oocytes were frozen immediately after injection. In some experiments, integration frequencies reached a plateau at 120 min, while in other experiments they continued to increase overnight (Fig. S1).

To determine whether second-strand synthesis could occur in *X. laevis* oocyte nuclei, a portion of the nucleic acids isolated at different time points was digested with the restriction enzymes KpnI or MluI, prior to transformation into *E. coli* for the Tet^R^ colony assay (Fig. 2C). KpnI and MluI have single cleavage sites within the intron and cleave double-stranded but not single-stranded DNA or RNA/DNA hybrids (verified by control experiments with DNA oligonucleotide substrates under the conditions used; Fig. S2). At the earliest time point (10 min), KpnI or MluI digestion had no significant effect on the recovery of Tet^R^ colonies, while at longer time points KpnI or MluI digestion virtually abolished the recovery of Tet^R^ colonies. By contrast, RNase H, which cleaves RNA in RNA/DNA heteroduplexes, or RNase A, which under low-salt conditions (50 mM NaCl) cleaves either single-stranded RNA or RNA in RNA/DNA heteroduplexes, had little if any effect (RNase A gave a<38% decrease at any time point in the experiment of Fig. 2C (not shown), and neither RNase A nor RNase H had a significant effect within experimental error in the experiment of Fig. S1). The specificities of RNase H and RNase A under the conditions used were also verified by control experiments with synthetic oligonucleotide substrates (Fig. S2). The finding that the intron-integration products isolated from *X. laevis* oocyte nuclei at longer time points are almost completely sensitive to restriction enzymes that cut within the intron but insensitive to RNases H or A indicates that the integrated intron had been largely converted to double-stranded DNA, presumably with the help of host enzymes for second-strand DNA synthesis (see Introduction).

### Group II intron-stimulated homologous recombination

**Figure 3:**
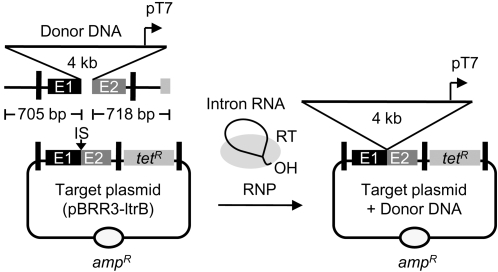
Assay of group II intron-RNP stimulated homologous recombination in *X. laevis* oocyte nuclei. A target plasmid (pBRR3-ltrB) containing the Ll.LtrB intron-insertion site (IS; ligated *ltrB* exon 1 and 2 sequences; E1 and E2) was co-injected into *X. laevis* oocyte nuclei with a 5.4-kb linear donor DNA, consisting of a 4-kb phage λ sequence with an inserted T7 promoter, flanked by 705- and 718-bp sequences homologous to those flanking the Ll.LtrB-insertion site in the target plasmid. Ll.LtrB RNPs containing the 0.9-kb Ll.LtrB-ΔORF intron and the group II intron RT were then injected into the same oocyte nuclei. A double-strand break resulting from intron RNA reverse splicing and second-strand cleavage at the Ll.LtrB target site stimulates homologous recombination, resulting in the insertion of the donor DNA containing the T7 promoter, thereby activating the *tet^R^* gene. Nucleic acids were isolated and electroporated into *E. coli* HMS174(DE3), and the targeting efficiency was calculated as the ratio of (Tet^R^+Amp^R^)/Amp^R^ colonies.

We showed previously that the Ll.LtrB group II intron could be used in *E. coli* to generate a targeted double-strand break that stimulates gene targeting by homologous recombination with a co-transformed DNA fragment by at least one to two orders of magnitude [8]. To test whether the Ll.LtrB intron could be used to introduce a recombinogenic double-strand break in *X. laevis* oocyte nuclei, we developed the assay shown in Figure 3. In this assay, the closed-circular target plasmid (pBRR3-ltrB), containing the Ll.LtrB intron-insertion site (IS), was co-injected with a 5.4-kb linear donor DNA and 500 mM MgCl_2_. The donor DNA consists of ∼4-kb of unrelated DNA sequence (3,967-bp phage λ DNA NcoI fragment) with a T7 promoter flanked by 705- and 718-bp homology arms corresponding to sequences on either side of the intron-insertion site in the recipient plasmid. After injecting the DNAs, we injected Ll.LtrB-ΔORF lariat RNPs, without an internal T7 promoter, into the same oocyte nuclei. Introduction of a double-strand break at the Ll.LtrB target site by the intron RNPs is expected to stimulate homologous recombination, resulting in insertion of donor DNA containing the T7 promoter upstream of the promoterless *tet^R^* gene. After incubating the oocytes, nucleic acids were isolated and electroporated into *E. coli*, and the recombination (“targeting”) efficiency was measured by the ratio of (Tet^R^+Amp^R^)/Amp^R^ colonies, as in the group II intron-integration assays.

As summarized in Table 2, the targeting efficiency in the presence of RNPs was 2.2% compared to 0.01% in the absence of RNPs, a greater than 200-fold stimulation (In other experiments, there was no detectable background of recombination in the absence of RNPs). The expected recombinant plasmid product obtained in the presence of RNPs was confirmed by sequencing the 3′-integration junction in plasmid DNAs from eight Tet^R^ colonies (not shown). In other experiments under the same conditions, targeting efficiencies were typically 0.3–1.5% with a maximum of 4.8%. By comparison, target plasmid that had been pre-cut with a restriction enzyme and tested in the same assay without RNPs gave a recombination frequency of 66% (Table 2). As expected, no Tet^R^ colonies were detected if the RNPs had been heat-inactivated at 95°C for 3 min, nor if either the donor DNA or the target plasmid were omitted from the assay (Table 2). Further, donor DNA that had not been linearized did not give detectable recombination (<0.005%), as expected for the single-strand annealing recombination pathway, which predominates in *X. laevis* oocyte nuclei and requires free ends on both the donor and recipient [50], [51]. As for site-specific group II intron integration, the group II intron-stimulated homologous recombination reaction was strongly dependent upon the injection of additional Mg^2+^, with recombination in the absence of injected Mg^2+^ falling to undetectable levels (<0.007%; Table 2). In the presence of Mg^2+^, addition of dNTPs did not significantly increase the targeting efficiency (not shown). Together, these findings show that Ll.LtrB RNPs can be used in *X. laevis* oocyte nuclei to introduce a targeted double-strand break that stimulates gene targeting by homologous recombination.

**Table 2:** Group II intron-stimulated homologous recombination in *X. laevis* oocyte nuclei.

Condition	Amp^R^+Tet^R^	Amp^R^	Targeting (%)
WT	2.2×10^2^	9.9×10^3^	2.2
-RNP	1.6	1.6×10^4^	0.01
Heated RNP	0	1.6×10^4^	<0.006
-Donor DNA	0	4.2×10^3^	<0.02
-MgCl_2_	0	1.5×10^4^	<0.007
Precut TP	8.6×10^2^	1.3×10^3^	66
Circular Donor DNA	0	2.1×10^4^	<0.005

Target plasmid pBRR3-ltrB (TP; 4.1 ng) and linear donor DNA (17 ng) were injected together into *X. laevis* oocyte nuclei in 17 nl of a solution containing 500 mM MgCl_2_, followed within 1 min by injection of Ll.LtrB RNPs (34 ng, 17 nl) reconstituted with Ll.LtrB lariat RNA without a T7 promoter in DIV. After a 2-h incubation at 25°C, nucleic acids were extracted from 10 pooled oocytes for each condition and electroporated into *E. coli* HMS174(DE3). In one sample, the target plasmid was precut with BtrI 27-bp upstream of the intron target site and injected without group II intron RNPs. Targeting frequencies were determined by the ratio of (Tet^R^+Amp^R^)/Amp^R^ colonies, as in intron-integration assays (Fig. 1 and Materials and Methods). Experiments for each condition except precut target plasmid DNA were repeated at least once with similar results.

### Biochemical activities of group II intron RNPs containing linear instead of lariat intron RNA

We wished to test whether RNPs reconstituted with linear intron RNA could be used to introduce the recombinogenic double-strand break at the target site. The rationale was that while linear intron RNA cannot undergo both transesterification reactions required for full reverse splicing, it is expected to be fully competent to carry out the first transesterification, *i.e.*, ligation of the 3′ end of the intron RNA to the 5′ end of the 3′ exon, leaving a DNA-strand break [52]. The use of linear intron RNA, which can be obtained directly by *in vitro* transcription without the self-splicing step required to generate lariat RNA, would facilitate the preparation of RNPs.

**Figure 4:**
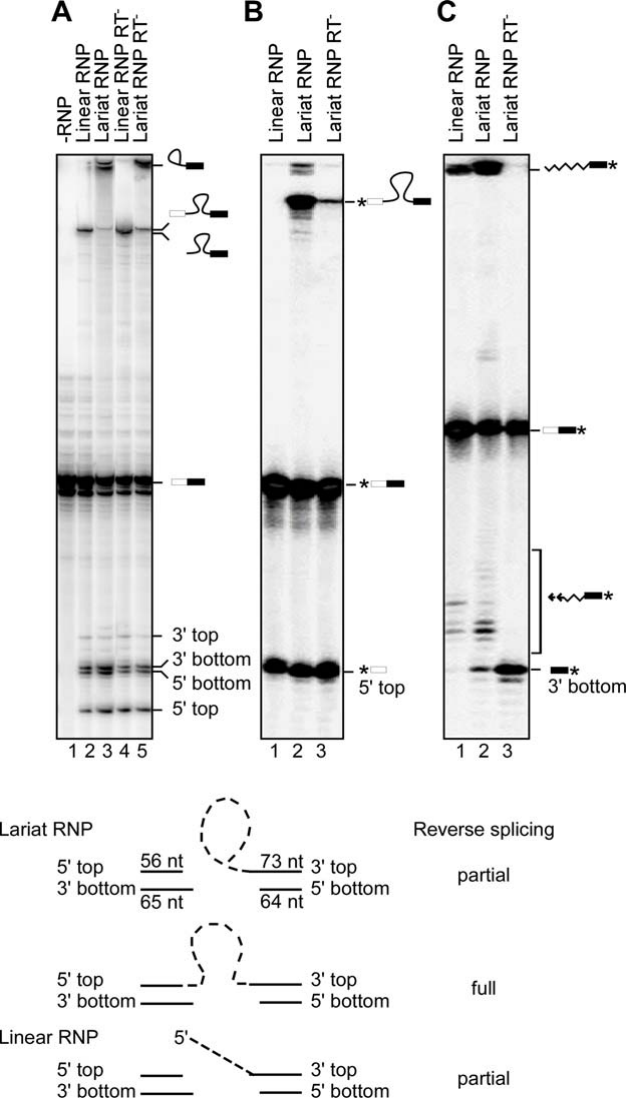
Target DNA cleavage and target DNA-primed reverse transcription reactions of RNPs reconstituted with lariat or linear Ll.LtrB intron RNA. Ll.LtrB RNPs containing lariat or linear intron RNA were incubated with ^32^P-labeled DNA oligonucleotide substrates containing the Ll.LtrB target site (positions −56 to +73 from the Ll.LtrB intron-insertion site), and the products were analyzed in a denaturing polyacrylamide gel. (A) Lariat and linear RNPs were incubated with internally labeled DNA substrate for 30 min at 37°C, as described in Materials and Methods. (B) and (C) Lariat and linear RNPs incubated with DNA substrates labeled (asterisk) at the 5′-end of the top and bottom strand, respectively in the presence of 0.2 mM dATP, dCTP, dGTP, and dTTP for 30 min at 37°C. In (A)–(C), products are indicated to the right of the gel. The schematic at the bottom diagrams the products expected for each reaction. All lanes are from the same gel, but some lanes in (A) were rearranged to appear adjacent.

First, we carried out biochemical assays to confirm that Ll.LtrB RNPs reconstituted with linear intron RNA could carry out the expected reactions. Figure 4A shows an experiment in which linear and lariat intron RNPs were incubated *in vitro* with an internally labeled double-stranded DNA oligonucleotide substrate containing the Ll.LtrB-insertion site, and the products were analyzed by electrophoresis in a denaturing polyacrylamide gel. As found previously [53], the RNPs containing lariat RNA gave products resulting from both full and partial reverse splicing of the intron RNA into the top strand, along with two closely spaced fragments resulting from IEP cleavage of the bottom strand (Fig. 4A, lane 3). By contrast, the RNPs containing linear intron RNA did not yield the same fully reverse-spliced product and intermediate containing attached lariat RNA as did lariat RNPs, but did cleave the top strand by partial reverse splicing, resulting in a small band corresponding to the 5′ exon (5′ top) and a high-molecular weight product containing linear intron RNA attached to the 5′ end of the 3′ exon (Fig. 4A, lane 2). The RNPs containing linear intron RNA also carried out IEP cleavage of the bottom strand, producing the same two closely spaced fragments as lariat RNPs (5′ and 3′ bottom; Fig. 4A, lane 2).

Figure 4B and C show additional reactions in which RNPs containing linear or lariat intron RNA were incubated with DNA substrates labeled at the 5′ end of the top or bottom strand, respectively, in the presence of dNTPs to support target-DNA-primed reverse transcription. The reactions with the 5′-top-strand labeled substrate confirm that RNPs reconstituted with linear intron RNA cleave the top strand and show that the reaction can occur as efficiently as cleavage by lariat RNA (Fig. 4B, lanes 1 and 2). The reactions with 5′-labeled bottom strand show that the cleaved bottom strand can be used as a primer for reverse transcription of the linear intron, yielding higher molecular weight product with different lengths of attached cDNA (Fig. 4C, lanes 1 and 2). The gels also show that cDNA synthesis increases the proportion of fully reverse-spliced product obtained with lariat RNPs, presumably by pulling the unfavorable equilibrium for full reverse splicing toward completion (Fig. 4B, lane 2) [54], and that lariat or linear intron RNPs reconstituted with mutant LtrA protein that lacks RT activity can still carry out reverse splicing and top- and bottom-strand cleavage even though they cannot synthesize cDNA (Fig. 4A, lanes 4 and 5, and Fig. 4B and C, lanes 3). Most importantly for gene targeting, the biochemical experiments show that group II intron RNPs reconstituted with linear intron RNA can introduce a double-strand break resulting from top and bottom strand cleavage at the DNA target site.

### Group II intron-stimulated homologous recombination using RNPs reconstituted with linear intron RNA

Having demonstrated the appropriate reactions *in vitro*, we next compared the ability of RNPs reconstituted with linear or lariat intron RNA to promote gene targeting by group II intron-stimulated homologous recombination in *X. laevis* oocyte nuclei. Gene targeting was again assayed by recombination of a donor DNA containing a T7 promoter into the recipient plasmid target site to activate the downstream *tet^R^* gene. As shown in Table 3, with all RNP preparations diluted to 1.9 mg/ml based on O.D._260_ measurements, the RNPs containing linear intron RNA stimulated gene targeting by recombination at an efficiency of about one third that of RNPs reconstituted with lariat RNA (0.9 and 0.26%, respectively). This difference in efficiency is within the range of variation for different RNP preparations, but could also reflect that the linear intron RNA transcribed *in vitro* has heterogeneous 3′ ends due to imprecise transcription termination by T3 RNA polymerase (see Materials and Methods).

**Table 3:** Group II intron-stimulated homologous recombination in *X. laevis* oocyte nuclei with RNPs containing lariat or linear intron RNA and wild-type or RT-deficient intron-encoded protein.

Condition	Amp^R^+Tet^R^	Amp^R^	Targeting (%)
Lariat RNP	2.0×10^2^	2.2×10^4^	0.9
Linear RNP	91	3.4×10^4^	0.26
Lariat RNP-RT^−^	15	4.2×10^4^	0.03
Linear RNP-RT^−^	7.3	4.3×10^4^	0.017

Target plasmid pBRR3-ltrB (4.1 ng) and linear donor DNA (17 ng) were injected together into *X. laevis* oocyte nuclei in 16 nl of a solution containing 500 mM MgCl_2_, followed within 1 min by injection of Ll.LtrB RNPs (38 ng, 20 nl). RNPs were reconstituted with Ll.LtrB lariat or linear intron RNA without a T7 promoter in DIV and wild-type or RT-deficient mutant LtrA protein (RT^−^). After incubating for 2 h at 25°C, nucleic acids were extracted from 10 pooled oocytes for each condition and electroporated into *E. coli* HMS174(DE3). Targeting efficiencies were determined by the ratio of (Tet^R^+Amp^R^)/Amp^R^ colonies, as in intron-integration assays (Fig. 1 and Materials and Methods). The experiment was repeated three times with similar results.

We also tested if lariat and linear intron RNPs containing RT-deficient LtrA could be used to introduce the recombinogenic double-strand break. We thought that such RNPs might give higher targeting efficiencies because they can stimulate homologous recombination by cleaving the target site without being able to synthesize a cDNA into the site. Surprisingly, we found that the RT-deficient lariat and linear RNPs have greatly decreased targeting efficiencies (0.03 and 0.017%, respectively; Table 3). We confirmed by *in vitro* reverse splicing assays that the RT-deficient linear and lariat RNPs used in these experiments had double-strand DNA cleavage activity comparable to that of the wild-type RNPs (not shown; Fig. 4A shows the same for different RNP preparations). A possible explanation is that lariat or linear intron RNA attached to the 5′ end of the 3′ exon inhibits recombination by impeding strand resection, but cannot be removed efficiently in *X. laevis* oocyte nuclei unless cDNA occurs to make the RNA a substrate for an endogenous RNase H. Together, the above experiments indicate that RNPs reconstituted with lariat or linear intron RNA and RT-proficient LtrA protein are the preferred means of introducing the recombinogenic double-strand break in *X. laevis* oocyte nuclei.

### Effect of chromatinization on group II intron targeting reactions

Plasmid DNAs injected into *Xenopus* oocyte nuclei become chromatinized over 4 h [50]. As shown in Table 4, when we injected target plasmid DNA and incubated the oocytes for 4 h at room temperature to allow chromatinization prior to injecting Ll.LtrB RNPs, both the group II intron-integration and double-strand break-stimulated homologous recombination reactions were inhibited strongly (the site-specific integration efficiency decreased from 22 to 0.55% in Exp. 1 and from 8.9 to 0.28% in Exp. 2; and the double-strand break-stimulated homologous recombination efficiency decreased from 2.1% to 0.04% in Exp. 3). In all cases, the persistence of target plasmid DNA in the oocyte nuclei was verified by the recovery of large numbers of Amp^R^ colonies in the chromatinized samples (Table 4). The residual site-specific intron integration and recombination reactions after chromatinization could reflect activity of the RNPs on chromatinized DNA or that some proportion of the plasmid target sites had not been chromatinized. The histone deacetylase inhibitor trichostatin A (TSA) added to the oocyte incubation medium before injection of the target plasmid resulted in at most a small increase in the intron-integration or homologous recombination efficiencies (Table 4). In other experiments, similar results were obtained with 50 mM sodium butyrate, another histone deacetylase inhibitor (not shown). These findings indicate that chromatin poses a significant barrier to group II intron gene targeting reactions, presumably because it impedes access of group II intron RNPs to their DNA target sites.

**Table 4:** Effect of chromatinization of the target plasmid on group II intron site-specific DNA integration and group II intron-stimulated homologous recombination in *X. laevis* oocyte nuclei.

Condition	Amp^R^+Tet^R^	Amp^R^	Targeting (%)
Exp. 1			
TP+RNP	4.8×10^2^	2.2×10^3^	22
cTP+RNP	242	4.4×10^4^	0.55
cTP+RNP+TSA/16 mM	139	2.6×10^4^	0.53
cTP+RNP+TSA/64 mM	166	1.7×10^4^	0.98
Exp. 2			
TP+RNP	2.5×10^3^	2.8×10^4^	8.9
cTP+RNP	97	3.4×10^4^	0.28
cTP+RNP+TSA/64 mM	121	2.3×10^4^	0.53
cTP+RNP+TSA/320 mM	101	2.0×10^4^	0.5
Exp. 3			
TP+RNP+Donor DNA	45	2.1×10^3^	2.1
cTP+RNP+Donor DNA	8.7	2.2×10^4^	0.04
cTP+RNP+Donor DNA+TSA/16 mM	6	2.4×10^4^	0.02
cTP+RNP+Donor DNA+TSA/64 mM	8	1.1×10^4^	0.07

Target plasmid (TP) pBRR3-ltrB (4.1 ng, 16 nl) was injected into *X. laevis* oocyte nuclei. In one trial (top row in each experiment), the injection of target plasmid was followed within 1 min by the separate injection of 500 mM MgCl_2_/17 mM dNTPs and Ll.LtrB RNPs, while in other trials, the oocytes were incubated for 4 h at room temperature to allow chromatinization of the target plasmid (cTP) prior to injection of MgCl_2_/17 mM dNTPs and RNPs. RNPs in experiments 1 and 2 were reconstituted with Ll.LtrB lariat RNA containing a T7 promoter in DIV, while in experiment 3, RNPs were reconstituted with Ll.LtrB lariat RNA without a T7 promoter. When used, trichostatin A (TSA) was added to the medium at the indicated concentration just prior to injection of the target plasmid. Ten oocytes were injected for each condition. After overnight incubation at 25°C, nucleic acids were extracted, electroporated into *E. coli* HMS174(DE3), and assayed for targeting by determining the ratio of (Tet^R^+Amp^R^)/Amp^R^ colonies, as described in Figure 1 and Materials and Methods. Experiments 1 and 2 are independent repeats with different batches of oocytes, while Experiment 3 was done with the same batch of oocytes as Experiment 1. A repeat of group II intron-stimulated homologous recombination with a different batch of oocytes gave similar results for unchromatinized and chromatinized target plasmid (targeting efficiencies 1.15 and 0.06%, respectively).

### Mg^2+^-dependent group II intron integration into plasmid target sites in *Drosophila melanogaster* and zebrafish embryos

The effects of chromatinization are expected to be mitigated during DNA replication, as occurs in developing embryos. To test whether the group II intron-integration reaction could occur in *D. melanogaster* and zebrafish embryos, we carried out RNP microinjection experiments using the same plasmid-based intron-integration assay used in *X. laevis* oocyte nuclei (see Fig. 1). For *Drosophila*, the target plasmid DNA with or without extra Mg^2+^ and group II intron RNPs were injected sequentially into the posterior of precellular blastoderm stage embryos, and for zebrafish, they were injected sequentially into the cytoplasm of one-cell embryos.

As shown in Table 5, we observed reasonably efficient Mg^2+^-dependent group II intron integration into the plasmid target site in both *D. melanogaster* and zebrafish embryos, as measured by the recovery of Tet^R^+Amp^R^ plasmids in *E. coli.* In both organisms, precise group II intron integration at the target site was confirmed by PCR and sequencing both the 5′- and 3′-integration junctions (not shown), and we observed little or no group II intron integration if extra MgCl_2_ was not injected with the target plasmid. For *D. melanogaster*, where the injection volumes were ∼0.3 nl, we obtained the highest integration efficiencies by injecting 250 mM MgCl_2_ with the target plasmid. Assuming an embryo volume of 7.3 nl [55], the calculated increase in intracellular Mg^2+^ resulting from this injection would be ∼10 mM. For zebrafish, where the injection volumes were ∼10 nl, the highest integration efficiencies were obtained by injecting 150 mM MgCl_2_, which assuming an embryo volume of 200 nl [56], corresponds to a calculated increase of intracellular Mg^2+^ of 7.5 mM or higher if one corrects for the 77% of embryo volume made up by the less permeable yolk. Thus, in both *Drosophila* and zebrafish embryos, the calculated increase in intracellular Mg^2+^ concentrations required to give maximal Ll.LtrB intron integration are in the same range as that found for *X. laevis* oocytes (∼9 mM). These findings suggest that a similar requirement for additional Mg^2+^ for maximal group II intron RNP integration may be a common feature of eukaryotic organisms.

**Table 5:** Plasmid assays for site-specific group II intron integration in *D. melanogaster* and zebrafish embryos.

Condition	Amp^R^+Tet^R^	Amp^R^	Integration (%)
*D. melanogaster*			
RNP	0	2.1×10^5^	0
RNP+TP+0.5 M MgCl_2_	8.4×10^3^	3.2×10^5^	2.7
RNP+TP+0.25 MgCl_2_	4.0×10^4^	4.2×10^5^	9.5
Zebrafish			
RNP	6	2.2×10^4^	0.027
RNP+TP+0.125 M MgCl_2_	1.1×10^2^	4.4×10^3^	2.5

Site-specific integration of group II intron RNPs into a plasmid target site in *D. melanogaster* and zebrafish embryos was assayed as described in Figure 1 and Materials and Methods. In the *D. melanogaster* experiment, target plasmid (TP) with or without MgCl_2_ and Ll.LtrB lariat RNPs were injected separately into the posterior of 100 precellular blastoderm stage embryos, which were then incubated for 1 h at 30°C. In the zebrafish experiment, target plasmid with or without MgCl_2_ and Ll.LtrB lariat RNPs were injected separately into the cytoplasm of 10 one-cell embryos and incubated for 1 h at 30°C. After incubation, nucleic acids were isolated and electroporated into *E. coli* HMS174(DE3), and the integration efficiency was determined as the ratio of (Tet^R^+Amp^R^)/Amp^R^ colonies. Qualitatively similar results were obtained in two independent repeats of the *Drosophila* experiment with different batches of RNPs (integration efficiencies 1.2 and 1.8% with 0.25 M or 0.5 M MgCl_2_ and zero without MgCl_2_) and in three independent repeats of the zebrafish experiment (integration efficiencies 0.56, 0.63, and 0.82% with 0.125 M MgCl_2_ and 0 to 0.004% without MgCl_2_).

For zebrafish embryos, hatch rates after the double injection of 500 mM MgCl_2_ and RNPs were 27–52% compared to 35% for a single injection of distilled water. For *D. melanogaster* embryos, the hatch rate after a single injection of 100 mM MgCl_2_, a concentration that supports chromosomal gene targeting (see below), was 69%. However, a double injection of 100 mM MgCl_2_ and RNP buffer decreased the hatch rate to 31% (not shown). Thus, both *D. melanogaster* and zebrafish appear reasonably tolerant of the high Mg^2+^ concentrations required to support group II intron integration, but the double injection appears better tolerated in zebrafish than in the smaller *D. melanogaster* embryos. We did not notice any further decrease in embryo viability caused by the injection of RNPs beyond that caused by the injections of Mg^2+^ and RNP buffer. We note, however, that toxicity could be greater at higher RNP concentrations or with RNPs targeted to different sites and will need to be assessed carefully in each case.

### Site-specific integration into chromosomal DNA target sites in *D. melanogaster* embryos

**Figure 5:**
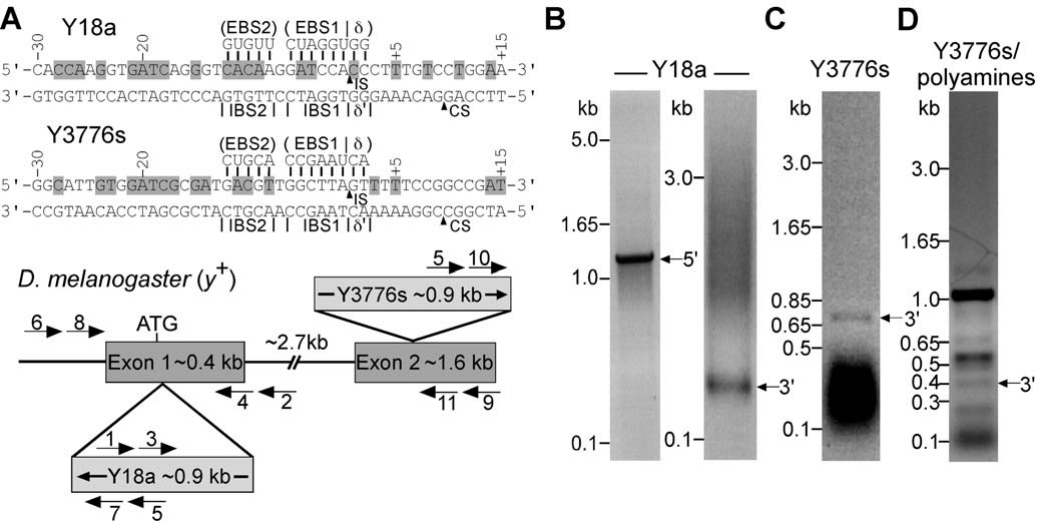
Site-specific integration of retargeted Ll.LtrB introns into chromosomal target sites in the *Drosophila melanogaster yellow* (*y*) gene. (A) DNA target site sequences and Ll.LtrB intron RNA base-pairing interactions for targetrons Y18a and Y3776s. Retargeted Ll.LtrB-ΔORF introns (targetrons) are denoted by a number that corresponds to the nucleotide position 5′ to the Ll.LtrB intron-insertion site numbered from the A of the ATG initiation codon, followed by “a” or “s”, indicating sense and antisense strand respectively. The DNA target sequences are shown from positions −30 to +15 from the intron-insertion site, with nucleotide residues that match those in the wild-type Ll.ltrB intron target sequence [7] highlighted in gray in the top strand. The intron-insertion site (IS) in the top strand and the IEP-cleavage site (CS) in the bottom strand are indicated by arrowheads. Below is shown a schematic of the *Drosophila yellow* (*y*) gene (NCBI accession number P09957), with the targetron-insertion sites indicated, and a diagram of the PCRs used to detect site-specific targetron insertion. *y* gene exons are gray rectangles, and introns and flanking sequences are lines. PCR primers used to detect and sequence the targetron integrations are indicated by numbered arrows. (B) PCR analysis of targetron Y18a integration using a double-injection protocol. Fifty embryos were injected with a solution containing 100 mM MgCl_2_+17 mM dNTPs followed by Y18a lariat RNPs (0.9 mg/ml). The embryos were incubated for 1 h at 30°C followed by 48 h at 18°C. Nucleic acids were isolated, and PCR products corresponding to the 5′- and 3′-junctions of Y18a integrated at its chromosomal target site (1,156-bp and 238-bp, respectively) were detected by nested PCR using the following primer pairs: 5′-junction, primers 1 (LtrB+940a) and 2 (yellow+277a), followed by primers 3 (LtrB+933a) and 4 (yellow+241a); 3′-junction, primers 5 (LtrB+788s) and 6 (yellow-350a), followed by primers 7 (LtrB+880s) and 8 (yellow-160s) (see Materials and Methods). (C) PCR analysis of targetron Y3776s integration using a double-injection protocol. Forty embryos were injected with 100 mM MgCl_2_ followed by Y3776s lariat RNPs (1.3 mg/ml), and the embryos were then incubated for 30 min at 37°C. Nucleic acids were isolated, and a 702-bp PCR product corresponding to the 3′-junction of Y3776s integrated at its target site was detected by PCR using primers 5 (LtrB+788s) and 9 (yellow+4325a) (see Materials and Methods). The dark band at the bottom of the gel is RNA. (D) PCR analysis of Y3776s integration using a single-injection protocol. Thirty embryos were injected with a solution containing100 mM KCl, 5 mM putrescine dihydrochloride, 3 mM spermidine trihydrochloride, 1 mM spermine tetrahydrochoride, 5 mM MgCl_2_, and Y3776s linear RNPs (0.5 mg/ml). After incubating the embryos for 30 min at 37°C, nucleic acids were isolated as described above, and a 348-bp PCR product (arrow) corresponding to the 3′-junction for Y3776s integrated at its target site was detected by nested PCR using primers 5 (LtrB+788s) and 9 (yellow+4325a), followed by primers 10 (LtrB+870s) and 11 (yellow+4054a) (see Materials and Methods). Other bands in the gel are likely due to non-specific annealing of the primers. In (B–D), the PCR products were analyzed by electrophoresis in a 1% agarose gel, which was stained with ethidium bromide, and the identities of the PCR products were confirmed by sequencing across the integration junctions (not shown).

Finally, we used the *D. melanogaster* embryos to test whether the reaction conditions we developed in plasmid targeting assays could also be used for site-specific integration into chromosomal target sites. For this purpose, we used retargeted Ll.LtrB introns (“targetrons”) designed to insert at two different sites in the *yellow* (*y*) gene (Fig. 5A). These targetrons are denoted Y18a and Y3776s, where the number corresponds to the nucleotide residue 5′ to their insertion site in the target gene numbered from the “A” of the ATG initiation codon, and “a” or “s” indicate antisense and sense strand, respectively.

In the first experiment (Fig. 5B), *D. melanogaster* embryos at the precellular blastoderm stage were injected with 100 mM Mg^2+^ plus 17 mM dNTPs, followed by Y18a lariat RNPs. After incubation for 1 h at 30°C followed by 48 h at 18°C, nucleic acids were extracted from 50 pooled embryos and analyzed by PCR. The PCRs showed bands corresponding to the expected 5′- and 3′-integration junctions (Fig. 5B), and precise integration at the correct site in the *y* gene was confirmed by sequencing the PCR products (not shown). A similar double-injection experiment showed precise integration of Y3776s lariat RNPs at their target site, detected by PCR and confirmed by sequencing of the 3′-integration junction in 40 pooled embryos. For both targetrons, integration into the chromosomal target sites was not detected without injection of Mg^2+^.

The decreased viability caused by double injection (see above) made it desirable to develop single-injection conditions. As indicated previously, however, group II intron RNPs tend to aggregate when injected with the relatively high concentrations of Mg^2+^ required for efficient DNA integration. Thus, we tested whether substitution of polyamines might enable us to lower the amount of Mg^2+^ that must be injected with the RNPs. Polyamines had been shown previously to reduce the Mg^2+^ requirement for group II intron self-splicing reactions [57]. Figure 5D shows that a single injection of linear Y3776s RNPs with 5 mM Mg^2+^ and a polyamine cocktail containing 5 mM putrescine, 3 mM spermidine, 1 mM spermine, corresponding to polyamine ratios in *D. melanogaster* larvae [58], gave site-specific integration into the *y* gene, detected by PCR in 30 pooled embryos and confirmed by sequencing the 3′-integration junction. Viability as measured by hatch rate after injection of this polaymine/Mg^2+^ mixture was similar to that for a single injection of distilled water. Thus, the use of polyamines to reduce the Mg^2+^ requirement for group II intron function provides a potential solution to the problem of double injection into *D. melanogaster* embryos, enabling the introduction of RNPs and required supplements in a single injection that gives chromosomal gene targeting.

## Discussion

Here, by using group II intron RNP microinjection assays, we show that group II intron-based gene targeting reactions can occur efficiently in eukaryotes, but are dependent upon the injection of additional Mg^2+^. First, we demonstrated Mg^2+^-dependent group II intron integration promoted by RNPs into plasmid target sites in *X. laevis* oocyte nuclei and used the facile *X. laevis* system to optimize reaction conditions. Then, by using these conditions, we showed similar Mg^2+^-dependent group II intron integration into plasmid target sites in both *Drosophila* and zebrafish embryos, and in *Drosophila*, we extended the methods to demonstrate site-specific integration into chromosomal target sites. We also show that with the injection of additional Mg^2+^, group II intron RNPs injected into *X. laevis* oocyte nuclei can be used to introduce a targeted double-strand break that stimulates gene targeting by homologous recombination. Thus, our results demonstrate that group II introns can potentially be used for two types of gene targeting reactions, site-specific integration and double-strand break-stimulated homologous recombination, in higher organisms.

The finding that Ll.LtrB intron RNP activity is limited by Mg^2+^ concentration in eukaryotes differs from the situation in bacteria, the natural hosts of group II introns, where free Mg^2+^ concentrations are apparently sufficient to support efficient mobility of Ll.LtrB and other group II introns. Group II introns seem to be particularly sensitive to low Mg^2+^ concentrations. Studies of yeast mtDNA group II introns showed that mutations in Mg^2+^ transporters that result in decreased intramitochondrial Mg^2+^ concentrations strongly inhibit the splicing of all four group II introns, which belong to different structural subclasses, while having relatively little effect on the splicing of group I introns [44], [45]. The group II introns affected by low Mg^2+^ concentrations in yeast mitochondria use different protein co-factors to promote RNA splicing [45], suggesting that the high Mg^2+^ requirement for group II intron function is a property of the intron RNA. For eukaryotic model organisms, the limitation of group II intron function by low Mg^2+^ concentrations can be overcome by injecting additional Mg^2+^ or Mg^2+^ in combination with polyamines. Longer term but more general solutions would be to select variants of the Ll.LtrB intron or to identify other group II introns that can function efficiently at lower Mg^2+^ concentrations.

Although group II intron RNPs can by themselves initiate site-specific DNA integration and double-strand break-stimulated recombination, host enzymes are required to complete both processes (see Introduction). An important finding here is that *X. laevis* oocyte nuclei appear to have the enzymatic machinery necessary to complete these reactions. Group II intron-stimulated homologous recombination is presumably completed by a single-strand annealing pathway, which has been shown previously to be active in *X. laevis* oocyte nuclei [51]. This pathway requires strand-resection by a 5′→3′ DNA endonuclease, strand annealing, and repair of gaps and nicks by DNA polymerase and DNA ligase activities. The only additional step required specifically for the group II intron-promoted reaction is the removal of the attached intron RNA from the 5′ end of the 3′ exon, which our findings for RT-deficient RNPs suggest may involve cDNA synthesis to make the RNA a substrate for endogenous RNase H [59] (Table 3). In the case of the site-specific DNA integration reaction, the late steps that require host enzymes likely include degradation of the intron RNA template strand by the endogenous RNase H, followed by second-strand DNA synthesis and DNA ligation, which again require DNA polymerase and DNA ligase activities. Previous studies of group II intron retrohoming showed that cDNA integration could occur by different DNA recombination or repair mechanisms in different organisms [16], [17], [60]. Collectively, these findings suggest that the availability of host enzymes to complete group II intron gene targeting reactions will not be a limitation in most systems.

The ability of group II introns to introduce a targeted double-strand break that stimulates gene targeting by homologous recombination was first demonstrated in bacteria [8]. However, prior studies in yeast mitochondria had shown that this mechanism is used naturally for the homing of mobile group II introns whose IEPs lack RT activity [61] and for the repair of abortive group II intron-integration events [62]. In the present work, we used a plasmid assay to characterize the group II intron-stimulated homologous reaction in *X. laevis* oocyte nuclei. A key finding is that group II intron RNPs reconstituted with linear as well as lariat intron RNA can be used to introduce the targeted double-strand break. This is so because although linear intron RNA is incapable of complete reverse splicing, it is fully competent to carry out the first step, attachment of the intron RNA to the 5′ end of the 3′ exon, leaving a DNA strand break. For both linear and lariat intron RNA, DNA cleavage occurs by a transesterification reaction with the attachment of the intron RNA to the target DNA and not by hydrolysis, a mechanism used for group II intron ribozyme cleavage of RNA substrates [63], [64]. The use of linear intron RNA facilitates the preparation of RNPs needed for gene targeting because it can be obtained directly by *in vitro* transcription without the self-splicing step needed for the preparation of lariat RNA.

In addition to their practical applications, our findings for RNPs containing linear intron RNA also have implications for group II intron mobility mechanisms. Some group II introns splice *in vivo* by using hydrolysis rather than branch-point formation for cleavage at the 5′ splice site, leading to the production of linear intron RNA [65]–[67]. Our findings suggest that such group II introns could be mobile by a mechanism involving partial reverse splicing into the target DNA, leading to attachment of linear intron RNA to the 5′ end of the 3′ exon, followed by second-strand cleavage and target DNA-primed reverse transcription of the linear intron RNA, provided that cellular enzymes could complete ligation of the cDNA into the target site.

Unsurprisingly, our results show that chromatinization of the plasmid DNA target site in *X. laevis* oocyte nuclei strongly inhibits both group II intron DNA integration and double-strand break-stimulated homologous recombination, presumably because it impedes access of the RNPs to the DNA target site. Although the inhibition by chromatinization appears greater for group II intron RNPs than was observed previously for the meganuclease I-SceI in analogous experiments in *X. laevis* oocyte nuclei [50], the two datasets are not strictly comparable without information about the degree of chromatinization of the different target sites. As for other gene targeting methods, interference from chromatin with group II intron gene targeting is expected to be mitigated in systems undergoing active transcription or DNA replication. Accordingly, we show here that microinjected group II intron RNPs promote efficient Mg^2+^-dependent plasmid targeting in both *Drosophila* and zebrafish embryos, and in the *Drosophila*, we show that optimal conditions developed in plasmid targeting assays can be used for site-specific integration into two different chromosomal target sites in the *y* gene. In both cases, the site-specific integration into chromosomal DNA occurred at frequencies high enough to detect by PCR and confirm by sequencing in 50 or less pooled embryos. Unlike previously developed *Drosophila* gene targeting methods [40], [68], the group II intron-integration method has the advantage of requiring only microinjection of RNPs and is not dependent upon prior strain construction and crosses to generate flies expressing DNA endonucleases and linear donor DNA.

Both group II intron integration and double-strand break-stimulated homologous recombination are potentially powerful methods for gene targeting in higher organisms. As retroelements akin to retroviruses, group II introns have a highly evolved DNA integration mechanism, which enables targeted rather than random integration. Further, we showed previously that mobile group II introns could be used as vectors to integrate genetic markers or cargo genes cloned in domain IV at desired DNA target sites in bacteria [5], [9], [43]. For applications in gene therapy, a potential advantage of the group II intron-integration reaction is that it can be accomplished by introduction of assembled RNPs without adding exogenous DNA that could result in deleterious ectopic integrations. For double-strand break-stimulated homologous recombination, the potential advantages of group II introns are that they have inherently very high target specificity and that the double-strand break can be targeted to different locations simply by modifying the base-pairing sequences in the intron RNA, without the need for protein engineering to change target specificity as for protein endonucleases. While further work will be required to develop the group II intron-based methods for routine use, our findings indicate that under appropriate conditions microinjected group II intron RNPs are highly active in eukaryotes, can access plasmid or chromosomal target sites, and can integrate or introduce double-strand breaks into those target sites at reasonably high efficiency. Thus, there appear to be no insurmountable barriers to using group II introns for gene targeting in eukaryotes.

## Materials and Methods

### Recombinant plasmids

pACD2 and pACD3 are intron-donor plasmids used for *in vitro* transcription of wild-type Ll.LtrB-ΔORF intron RNA, which is then reconstituted with purified LtrA protein to form RNPs (“targetrons”) for gene targeting [4], [8]. Both plasmids contain the 0.9-kb Ll.LtrB-ΔORF intron and flanking exons cloned downstream of a T7lac promoter in a pACYC184-based vector with a *cam^R^* gene, and have the LtrA ORF cloned downstream of the 3′ exon. In pACD2, the Ll.LtrB-ΔORF intron contains an additional phage T7 promoter inserted in intron domain IV (DIV) for use in plasmid-based DNA-integration assays. pACD4C and pACD4G are derivatives of pACD3, which have C or G at the δ′ +1 position in the 3′ exon and were used for construction and *in vitro* transcription of targetrons Y18a and Y3776s, respectively [7]. The latter are Ll.LtrB-ΔORF introns in which the EBS2, EBS1 and δ sequences were modified to be complementary to IBS2, IBS1, and δ′ sequences in DNA target sites in the *D. melanogaster yellow* (*y*) gene. The donor plasmid used for each targetron has a δ′ residue in the 3′ exon complementary to the retargeted δ residue in the intron RNA for optimal RNA splicing [7].

pBRR3-ltrB, the target plasmid for intron-integration assays, contains the Ll.LtrB homing site (ligated exon 1 and 2 of the *ltrB* gene from position −178 upstream to +91 downstream of the intron-insertion site) cloned upstream of a promoterless *tet^R^* gene in an Amp^R^ pBR322-based vector [4], [8].

pIMP-1P, used for expression of the LtrA protein, contains the LtrA ORF cloned downstream of a tac promoter and Φ10 Shine-Dalgarno sequence in the protein-expression vector pCYB2 (New England Biolabs, Ipswich, MA) [46]. LtrA is expressed from this plasmid as a fusion protein with a C-terminal tag containing an intein-linked chitin-binding domain, enabling LtrA purification via a chitin-affinity column, followed by intein-cleavage. pImp-10P-DD^−^ expresses RT-deficient LtrA (RT^−^) with the mutation YADD→YAAA at the RT active site [69].

pUC19-PUF, used to generate the linear donor DNA for group II intron-stimulated homologous recombination, contains homology arms of 705- and 718-bp upstream and downstream of the Ll.LtrB-insertion site, respectively, flanking a 3,967-bp phage λ DNA NcoI fragment and a T7 promoter sequence. To construct this plasmid, the left and right homology arms were amplified from pBRR3-ltrB with primers pBRR-700BamS (5′-CGGC*GGATCC*TTCTTCGGGGCGAAAACTCTCAAGG)+PUFNcoIT7A (5′-GAATTAAAAATGATATGCCCTATAGTGAGTCGTATTA *CCATGG*GTTATGGATGTGTTCAC) and PUFNcoIT7S (5′-GTGAACACATCCATAAC*CCATGG*TAATACGACTCACTATAGGGCATATCATTTTTAATTC)+pBRR+700BamA (5′-CGGC*GGATCC*CTGGGCGGCGGCCAAAGCGGTCGGA), respectively. The PUFNcoIT7A primer introduces a NcoI site (italics) and a phage T7 promoter (underlined), and the outside primers introduce BamHI sites (italics). The product obtained in a second PCR with the outside primers was cloned into the BamHI site of pUC19, and the 3,967-bp NcoI fragment of phage λ DNA was inserted into the NcoI site.

### Preparation and biochemical analysis of group II intron RNPs

Ll.LtrB-ΔORF intron RNPs were reconstituted from *in vitro*-synthesized intron RNA and purified LtrA protein by a modification of a method described previously [46]. A precursor RNA containing the Ll.LtrB-ΔORF intron and flanking exons was transcribed with phage T7 RNA polymerase (Megascript T7 Kit; Ambion, Austin, TX) from the appropriate pACD-based donor plasmid (see above) which had been linearized with NheI, or with phage T3 RNA polymerase (Megascript T3 Kit; Ambion) from a PCR product generated from the donor plasmid, using the 5′ primer pACD-T3 (5′ GGAGTCTAGAAATTAACCCTCACTAAAGGGAATTGTGAGCG), which appends a T3 promoter sequence (underlined), and the 3′ primer LtrB+744a (5′ CTCCTCTAGAATCCGCTGTATCATCTAATATTCCTTTTG) or NheIR (CTAGCAGCACGCCATAGTGACTGGCG). The resulting precursor RNAs containing the Ll.LtrB-ΔORF intron and flanking exon sequences were self-spliced in 1.25 M NH_4_Cl, 50 mM MgCl_2_, 50 mM Tris-HCl, pH 7.5 for 3 h at 37°C, then ethanol-precipitated and dissolved in distilled water.

To reconstitute Ll.LtrB-ΔORF RNPs, the self-spliced RNA (100 nM) was re-natured by heating to 50 or 55°C in 10 ml of 450 mM NaCl, 5 mM MgCl_2_, 40 mM Tris-HCl, pH 7.5 and slowly cooling to 30°C prior to addition of 200 nM of purified LtrA protein [46] and further incubation for 30 min at 30°C. The resulting RNPs were pelleted by ultracentrifugation in a Beckman 50.2 Ti rotor at 145,000×g for 16 h at 4°C and resuspended in 50 µl of 10 mM KCl, 10 mM MgCl_2_, and 40 mM HEPES, pH 8.0. The RNP preparations typically contain 60–70% intron lariat RNA with the remainder being precursor RNA plus smaller amounts of linear intron and ligated exons, and they typically have a concentration of 2–3 mg RNA/ml based on O.D._260_, with the highest obtained being 5.6 mg/ml.

To make RNPs containing linear intron RNA, Ll.LtrB-ΔORF intron RNA was transcribed with phage T3 RNA polymerase from a PCR product generated from pACD3 using the 5′ primer T3-LIs (AATTAACCCTCACTAAAGG G TGCGCCCAGATAGGGTGTTAAGTCAAG), which appends a T3 promoter (underlined), and the 3′ primer LtrB+940a (GTGAAGTAGGGAGGTACCGCCTTGTTC), which corresponds to the 3′ end of the intron. The linear RNA was renatured and reconstituted with purified LtrA protein [46] as described above for lariat RNPs.

DNA endonuclease assays were carried out with a 129-bp DNA substrate, which was internally labeled by PCR in the presence of [α-^32^P]dTTP (3,000 Ci/mmol; Perkin-Elmer, Waltham, MA) [53], or with 60-bp DNA oligonucleotide substrates labeled at the 5′ end of the top or bottom strand with [γ-^32^P]ATP and phage T4 polynucleotide kinase (New England Biolabs). The 60-bp DNA substrates were prepared by annealing complementary synthetic oligonucleotides and purified using a centri-sep spin column (Princeton Separations, Adelphia, NJ). For assays with the internally labeled DNA substrate, the DNA (1.5 nM; 56,000 cpm) was incubated with RNPs (815 nM) for 30 min at 37°C in 20 µl of reaction medium containing 10 mM KCl, 10 mM MgCl_2_, and 50 mM Tris-HCl, pH 7.5. For assays with the 5′-labeled DNA substrates, the DNA (50 nM) was incubated with RNPs (100 nM) for 30 min at 37°C in 20 µl of the same reaction medium plus 0.2 mM each of dATP, dCTP, dGTP, and dTTP (referred to as dNTPs). After the incubations, the reactions were terminated by extraction with phenol-chloroform-isoamyl alcohol (25:24:1; phenol-CIA) followed by ethanol precipitation, and the products were analyzed by electrophoresis in denaturing 6% or 10% (w/v) polyacrylamide gels, which were dried and scanned with a phosphorimager.

### *X. laevis* methods

Mature adult female frogs were obtained from Nasco (Fort Atkinson, WI). Stage VI oocytes were manually peeled from the follicle cell layer in isolation medium (108 mM NaCl, 2 mM KCl, 1 mM EDTA, 1 mM HEPES, pH 7.5), treated with 0.05% collagenase (Sigma-Aldrich, St. Louis, MO) for 10 min, and rinsed in incubation medium [Barth's solution (88 mM NaCl, 1 mM KCl, 0.82 mM MgSO_4_, 2.4 mM NaHCO_3_, 0.33 mM Ca(NO_3_)_2_, 0.91 mM CaCl_2_, 10 mM N-2-hydroxyethylpiperazine-N′-ethanesulphonic acid, pH 7.4) supplemented with penicillin (10,000 units/l), streptomycin (10 mg/l), gentamycin (50 mg/l), and theophylline (90 mg/l)]. Oocytes were kept in incubation medium at 16°C for at least 30 min before injection.

Oocyte nuclear injection was done in incubation medium using a pressure system (Picospritzer III; Parker Hannifin, Mentor, OH) with 20 psi output. The injection volume was calibrated to 10–20 nl for each needle. A micromanipulator (MN-151; Narishige, Tokyo, Japan) was used to manipulate the injection needles.

For plasmid targeting assays, the target plasmid DNA (4–10 mg/ml) with specified amounts of MgCl_2_ was injected into *Xenopus laevis* oocyte nuclei, followed within 1 min by the injection of RNPs (0.9–5.6 mg/ml), using different needles to avoid prior mixing. In most experiments, 17 mM each of dATP, dCTP, dGTP, and dTTP (dNTPs; Invitrogen, Carlsbad, CA; stocks concentrated to 70 mM using a Speed-Vac) was injected with the target plasmid. Integration efficiency increased with increasing RNP concentrations, but varied between different RNP preparations. For each experimental condition, 10 to 20 oocytes were injected and pooled for analysis. After injection, the oocytes were rinsed twice in incubation medium, incubated for times and temperatures specified for individual experiments, and stored at −80°C. For time-course experiments, the oocytes were quickly frozen in dry ice at the specified time after the last injection.

To isolate nucleic acids, the oocytes were thawed and incubated in lysis buffer [20 mM Tris-HCl, pH 8.0, 5 mM EDTA, 400 mM NaCl, 1% SDS (w/v), 400 µg/ml proteinase K (Molecular Biology Grade; Sigma-Aldrich)] for 1 h at 55°C, and then extracted twice with phenol-CIA. Nucleic acids were precipitated with isopropanol and dissolved in 20–50 µl of distilled water. Two µl of the nucleic acid preparation was electroporated into *E. coli* HMS174(DE3) F^−^, *hsdR, recA, rif* ^r^ (Novagen, EMD Chemicals, Gibbstown, NJ), and cells were plated at different dilutions on Luria-Bertani (LB) medium containing ampicillin (50 µg/ml) plus tetracycline (25 µg/ml) or the same concentration of ampicillin alone. Colonies were counted after overnight incubation at 37°C, and the integration efficiency was calculated as the ratio of (Amp^R^+Tet^R^)/Amp^R^ colonies. Site-specific intron integration was confirmed by PCR using primers For-pBRR (5′- CTGATCGATAGCTGAAACGC) and Rev2-pBRR (5′-AATGGACGATATCCCGCA) to amplify the inserted intron and flanking sequences, and then using Rseq (5′-CCATGCGAGAGTAGGGAAC) and Rev2-pBRR (see above) to sequence the 5′- and 3′-integration junctions, respectively.

For group II intron-stimulated homologous recombination reactions, *X. laevis* oocyte nuclei were injected with a mixture of closed-circular target plasmid pBRR3-ltrB (4–5 ng) and donor DNA (4–17 ng) in 10–20 nl of 0.5 M MgCl_2_, 10 mM Tris-HCl, pH 7.5, unless specified otherwise. The donor DNA was a 5.4-kb linear DNA generated by BamHI-digestion of pUC19-PUF (see above), followed by phenol-CIA extraction and ethanol precipitation. Ll.LtrB RNPs (0.9–5.6 mg/ml), without an internal T7 promoter, were injected into the same oocyte nuclei. Ten to twenty oocytes were injected and pooled for each experimental condition. The injected oocytes were incubated under conditions specified for individuals experiments, then nucleic acids were extracted and electroporated into *E. coli* HMS174(DE3), followed by plating on LB containing ampicillin with or without tetracycline, as for the group II intron-integration assays (see above).

### Zebrafish methods

Wild-type *D. rerio* strains were the AB line [70]. Zebrafish were reared at 28.5°C under a 14 h/10 h light/dark cycle, with *in vitro* fertilization performed, as described [71]. Fertilized eggs were incubated at room temperature for ∼15 min prior to injection. For group II intron targeting assays, target plasmid (0.5 mg/ml in a solution containing 3.125 mM of each dNTP and specified amounts of MgCl_2_, with 0.25% phenol red) and Ll.LtrB-ΔORF RNPs (0.1 mg/ml with 0.25% phenol red, 10 mM KCl, 10 mM MgCl_2_, and 40 mM HEPES, pH 8) were injected separately into 10 to 25 one-cell embryos in Steinberg's medium [72], using a pressure system (Picospritzer III; Parker Hannifin) with 20 psi output. The injection volumes were ∼10 nl. A micromanipulator (MN-151, Narishige) was used to manipulate the injection needles. After injection, the embryos were washed with Steinberg's medium, pooled in a single 1.5-ml Eppendorf tube in 500 µl Steinberg's medium, and incubated for 1 h at 30°C, prior to plasmid DNA extraction and transformation of *E. coli*, as described above for *X. laevis* assays.

### *D. melanogaster* methods

The *w* ^1118^ strain was grown in standard fly media at 22°C and embryos were collected on apple juice agar plates with yeast paste [73] for collection times not exceeding 40 min. The embryos were then bleach dechorionated, rinsed in egg wash [0.7% (w/v) NaCl, 0.04% (v/v) Triton X-100], adhered to a 25×75 mm slide, desiccated in a dish that contains Drierite and covered in Halocarbon oil (700 Series; Halocarbon Products, River Edge, NJ). Microinjection was done using filament needles with a bore size of ∼5 µm attached to an air-filled 50-ml syringe. A micromanipulator (MO-150; Narishige) was used to manipulate the injection needles. The injection volumes were ∼0.3 nl.

For plasmid-based group II intron-integration assays, target plasmid DNA (0.5 mg/ml) supplemented with 17 mM dATP, dCTP, dGTP, and dTTP (Invitrogen) and specified amounts of MgCl_2_ was injected into the posterior of the embryo, followed within 5 min by the injection of RNPs (1.6 mg/ml), using different needles to avoid prior mixing. For each experimental condition, 50 to 100 embryos were injected, incubated in a humidified chamber for 1 h at 30°C, taken up with a pipette, pooled, and homogenized in lysis buffer (see above) using a hand homogenizer. Plasmid DNAs were then extracted and transformed into *E. coli*, as described above for *X. laevis* assays.

For chromosome targeting, precellular blastoderm stage embryos (<40-min collection time) were injected with targetron Y18a or Y3776s RNPs, which contain Ll.LtrB introns targeted to insert at sites in the *y* gene. Mg^2+^, dNTPs, and polyamines were injected prior to or mixed with the RNPs, and after the injections, the embryos were incubated, as specified for individual experiments in the legend of Figure 5. Thirty to fifty embryos were then pooled, and nucleic acids were extracted, as described above. Targetron integrations were detected by PCR of one fifth of the extracted DNA using the following primers: Y18a, 5′-integration junction: primers LtrB+940a (5′-GTGAAGTAGGGAGGTACCGCCTTGTTC) and yellow+277a (5′-ACGATCTCCCCAAGGGCTTCAT) and nested primers LtrB+933a (5′-AGGGAGGTACCGCCTTGTTCACATTAC) and yellow+241a (5′-TACCATCACGCCAGCGGGGAA); Y18a, 3′-integration, LtrB+788s (5′-CGACTAATACGACTCACTATAGGGTC) and yellow-350s (5′-GCAAAGTTGGCCGATCTATGGGAAC) and nested primers LtrB+880s, (5′-GAAGAGGGTGGTGCAAACCAGTCAC) and yellow-160s, (5′-CGCCACGGTCCACAGAAGAG); Y3776s, 3′-integration junction: primers LtrB+788s (see above) and yellow+4325a (5′-ATGCCACCACCCAGATTGG) and nested primers LtrB+870s and yellow +4054a (5′-TGAGGTTTCTGTGGCAAGACAGGA). In each case, the PCR products were purified in a 1% agarose gel and sequenced to confirm integration at the expected target site.

### Treatment of animals

Animal care met the principles and guidelines of the Institute for Laboratory Animal Research “Guide for Care and Use of Laboratory Animals” and the University of Texas at Austin Institutional Animal Care and Use Committee.

## Supporting Information

Figure S1 Nuclease-sensitivity of group II intron-integration products extracted from *X. laevis* oocyte nuclei. Target plasmid DNA (4.5 ng/18 nl) with 500 mM MgCl_2_ and 17 mM dNTPs was injected into *X. laevis* oocyte nuclei, followed by Ll.LtrB lariat RNPs (53 ng/18 nl). The oocytes were incubated at 25°C for the indicated times and then quick frozen on dry ice. Nucleic acids were extracted, and equal portions were either untreated or digested in 10 µl reactions with MluI (10 units, 1 h at 37°C), RNase A (1 µl Sigma-Aldrich GenElute Mammalian Genomic DNA Miniprep Kit, 5 min at room temperature) or RNase H (0.05 units, 20 min at 37°C). MluI and RNase H digestions were done according to the manufacturers' protocols, and RNase A digestion was done in 50 mM NaCl, 10 mM Tris-HCl, pH 7.5. After digestion, the nucleic acids were extracted twice with phenol-CIA and ethanol precipitated in the presence of glycogen carrier prior to electroporation into *E. coli* HMS174(DE3). Integration efficiencies were determined as the ratio of (Tet^R^+Amp^R^)/Amp^R^ colonies. O/N, overnight.
(0.12 MB TIF)

Figure S2 Digestion of DNA and RNA oligonucleotide substrates with restriction enzymes and ribonucleases A and H. (A) Single-stranded (ss) DNA, DNA/RNA heteroduplex, and double-stranded (ds) DNA substrates (0.28 nM; 1,200–9,000 cpm) were incubated with MluI or KpnI (0, 10, 50, or 100 units; New England Biolabs) in 100-µl of reaction medium for 1 h at 37°C according to the manufacturer's protocol. (B) RNA, RNA/DNA heteroduplex, and double-stranded DNA substrates (0.28 nM; 1,200–9,000 cpm) were incubated in 100-µl reactions with RNase A (0, 0.2, 2, and 10 µl; Sigma-Aldrich GenElute Mammalian Genomic DNA Miniprep Kit) or RNase H (0, 0.5, and 50 units; New England Biolabs). RNase A digestion was done in 50 mM NaCl, 10 mM Tris-HCl, pH 7.5 for 5 min at room temperature, and RNase H digestion was done for 20 min at 37°C according to the manufacturer's protocol. In (A) and (B), substrates were DNA oligonucleotide 5′ TGAACAAGGCGGTACCTCCCTTGGCGACGCGTTGGGAAATGGCAATGATA by itself or annealed to a complementary DNA or RNA oligonucleotide, or the complementary RNA strand by itself. DNA and RNA oligonucleotides were obtained from Integrated DNA Technologies (Coralville, IA) and 5′-end labeled with [γ-^32^P]ATP (10 Ci/mmol; Perkin-Elmer) using phage T4 polynucleotide kinase (New England Biolabs) according to the manufacturer's protocol, then gel-purified in a denaturing 6% (w/v) polyacrylamide gel. In the ssDNA, DNA/RNA duplex, and dsDNA substrates, the DNA oligonucleotide was labeled, whereas in the RNA and RNA/DNA duplex substrates, the RNA oligonucleotide was labeled. Duplexes were formed by incubating the labeled oligonucleotide with an equal amount of unlabeled complementary strand, heating to 95°C and slow cooling to room temperature, then gel purifying in a 2% agarose gel containing Tris-borate-EDTA buffer (90 mM Tris, 90 mM boric acid, 2 mM EDTA). After incubation with the indicated enzymes, samples were extracted with phenol-CIA and ethanol precipitated in the presence of linear acrylamide carrier (58 µg/ml). Restriction enzyme digests were analyzed in a denaturing 8% (w/v) polyacrylamide gel, and RNase A and H digestions were analyzed in a denaturing 15% (w/v) polyacrylamide gel. Size markes were 5′-labeled 50- and 18-nt DNA oligonucleotides. The gels were dried and scanned with a phosphorimager.
(3.45 MB TIF)
