# Supplementary figures and images for: Group II Intron-Based Gene Targeting Reactions in Eukaryotes
Source: PLoS One. 2008 Sep 1;3(9):e3121. doi: 10.1371/journal.pone.0003121 (PMC2518211; doi:10.1371/journal.pone.0003121)

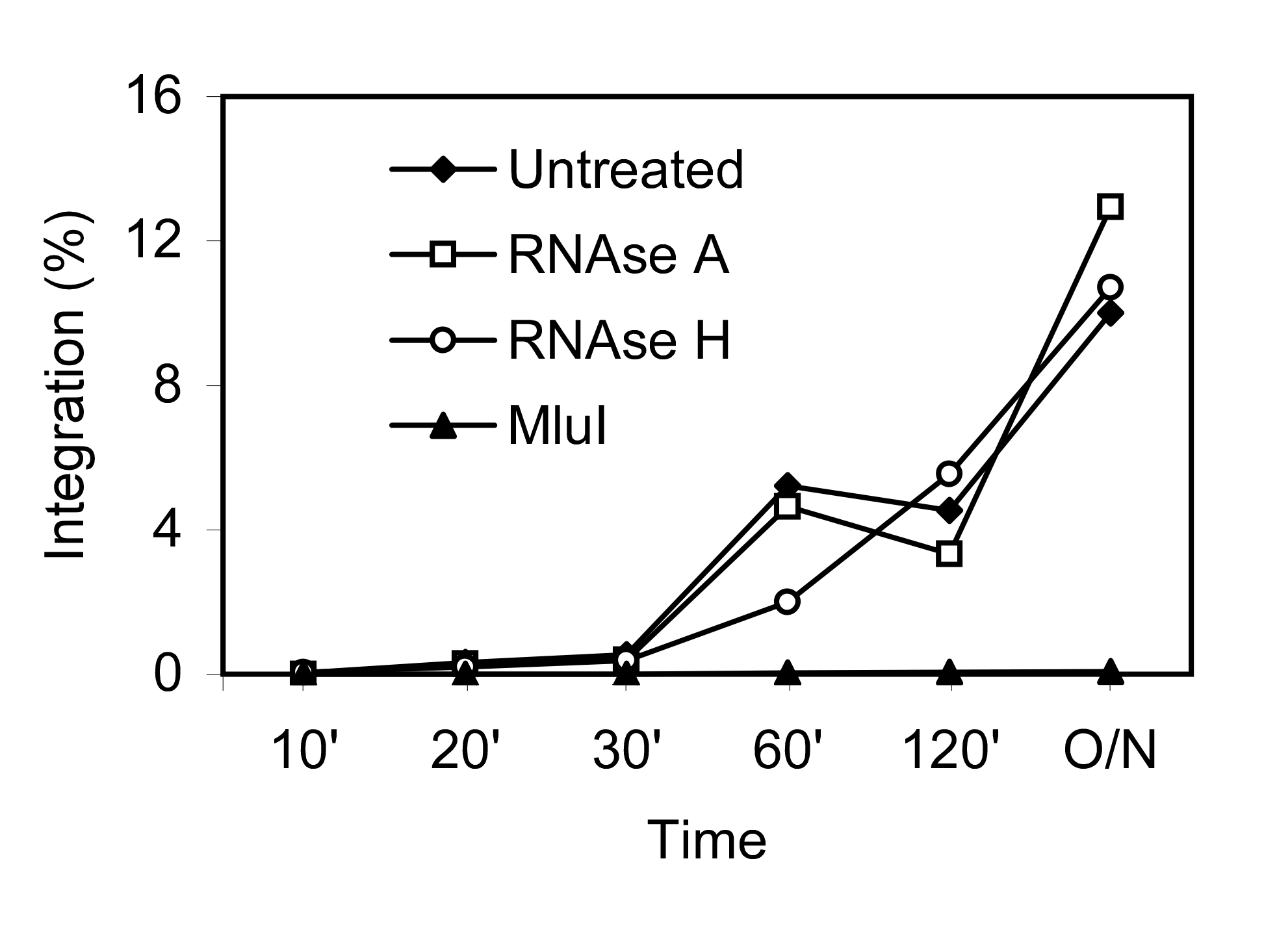

Supplement: Figure S1 — Nuclease-sensitivity of group II intron-integration products extracted from X. laevis oocyte nuclei. Target plasmid DNA (4.5 ng/18 nl) with 500 mM MgCl2 and 17 mM dNTPs was injected into X. laevis oocyte nuclei, followed by Ll.LtrB lariat RNPs (53 ng/18 nl). The oocytes were incubated at 25°C for the indicated times and then quick frozen on dry ice. Nucleic acids were extracted, and equal portions were either untreated or digested in 10 µl reactions with MluI (10 units, 1 h at 37°C), RNase A (1 µl Sigma-Aldrich GenElute Mammalian Genomic DNA Miniprep Kit, 5 min at room temperature) or RNase H (0.05 units, 20 min at 37°C). MluI and RNase H digestions were done according to the manufacturers' protocols, and RNase A digestion was done in 50 mM NaCl, 10 mM Tris-HCl, pH 7.5. After digestion, the nucleic acids were extracted twice with phenol-CIA and ethanol precipitated in the presence of glycogen carrier prior to electroporation into E. coli HMS174(DE3). Integration efficiencies were determined as the ratio of (TetR+AmpR)/AmpR colonies. O/N, overnight. (0.12 MB TIF) [file pone.0003121.s001.tif]

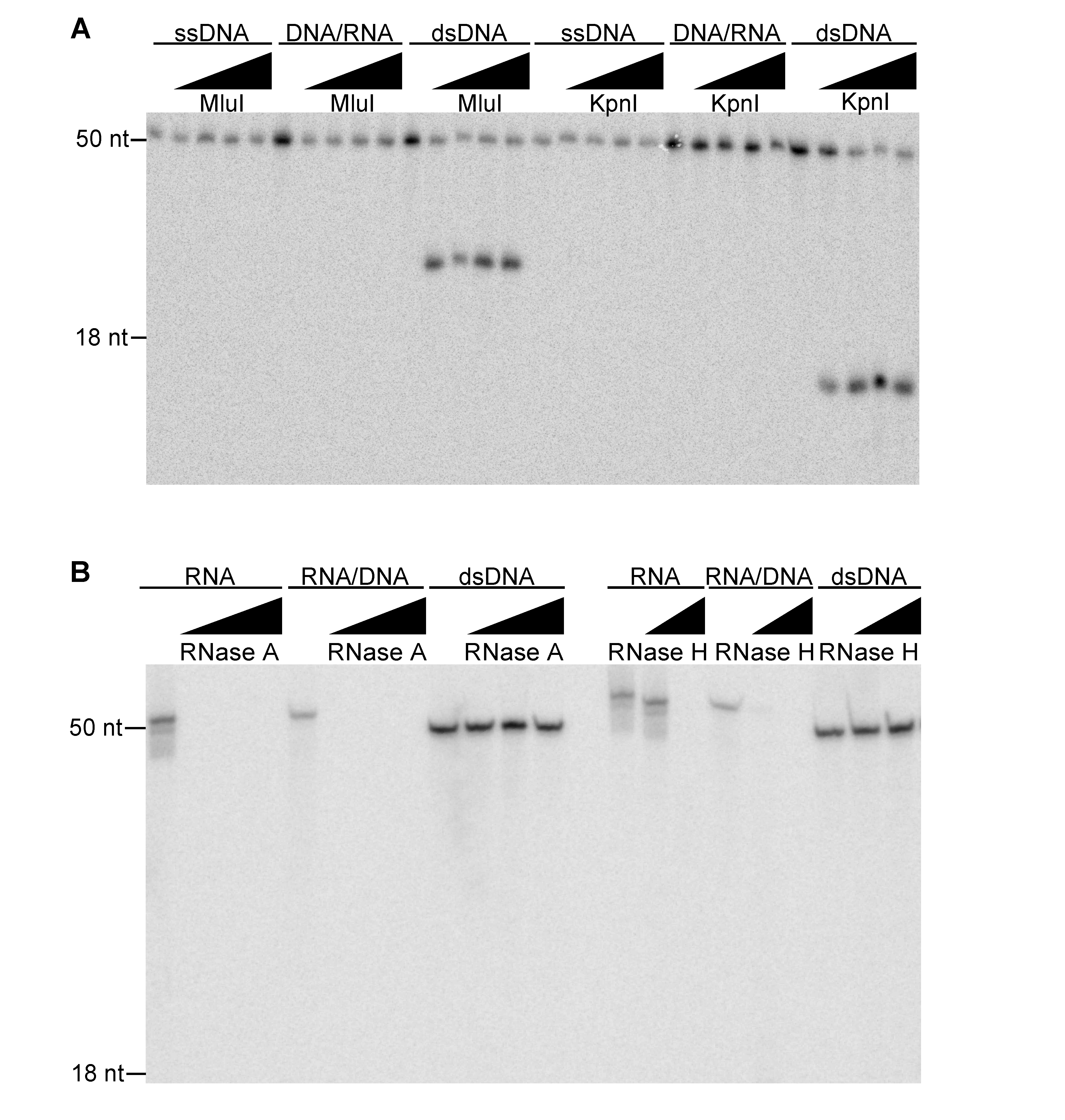

Supplement: Figure S2 — Digestion of DNA and RNA oligonucleotide substrates with restriction enzymes and ribonucleases A and H. (A) Single-stranded (ss) DNA, DNA/RNA heteroduplex, and double-stranded (ds) DNA substrates (0.28 nM; 1,200–9,000 cpm) were incubated with MluI or KpnI (0, 10, 50, or 100 units; New England Biolabs) in 100-µl of reaction medium for 1 h at 37°C according to the manufacturer's protocol. (B) RNA, RNA/DNA heteroduplex, and double-stranded DNA substrates (0.28 nM; 1,200–9,000 cpm) were incubated in 100-µl reactions with RNase A (0, 0.2, 2, and 10 µl; Sigma-Aldrich GenElute Mammalian Genomic DNA Miniprep Kit) or RNase H (0, 0.5, and 50 units; New England Biolabs). RNase A digestion was done in 50 mM NaCl, 10 mM Tris-HCl, pH 7.5 for 5 min at room temperature, and RNase H digestion was done for 20 min at 37°C according to the manufacturer's protocol. In (A) and (B), substrates were DNA oligonucleotide 5′ TGAACAAGGCGGTACCTCCCTTGGCGACGCGTTGGGAAATGGCAATGATA by itself or annealed to a complementary DNA or RNA oligonucleotide, or the complementary RNA strand by itself. DNA and RNA oligonucleotides were obtained from Integrated DNA Technologies (Coralville, IA) and 5′-end labeled with [γ-32P]ATP (10 Ci/mmol; Perkin-Elmer) using phage T4 polynucleotide kinase (New England Biolabs) according to the manufacturer's protocol, then gel-purified in a denaturing 6% (w/v) polyacrylamide gel. In the ssDNA, DNA/RNA duplex, and dsDNA substrates, the DNA oligonucleotide was labeled, whereas in the RNA and RNA/DNA duplex substrates, the RNA oligonucleotide was labeled. Duplexes were formed by incubating the labeled oligonucleotide with an equal amount of unlabeled complementary strand, heating to 95°C and slow cooling to room temperature, then gel purifying in a 2% agarose gel containing Tris-borate-EDTA buffer (90 mM Tris, 90 mM boric acid, 2 mM EDTA). After incubation with the indicated enzymes, samples were extracted with phenol-CIA and ethanol precipitated in the presence of linear acryl [file pone.0003121.s002.tif]
